# Supplementary material for: Macroeconomic impact of Ebola outbreaks in Sub-Saharan Africa and potential mitigation of GDP loss with prophylactic Ebola vaccination programs
Source: PLoS One. 2023 Apr 11;18(4):e0283721. doi: 10.1371/journal.pone.0283721 (PMC10089322; doi:10.1371/journal.pone.0283721)
Supplement: S1 Table — DRC, democratic republic of the Congo; GDP, gross domestic product. (DOCX) [file pone.0283721.s001.docx]

**­­­­­S1 Table. Pre-Outbreak GDP per Capita of Ebola-affected Countries and Prediction Error: Synthetic Control Versus the Average of Donor Countries and the Two Most Similar Countries.**

| **Country** | **Year** | **Actual** | **Pre-outbreak Prediction Errors** | | | | | | | |
| --- | --- | --- | --- | --- | --- | --- | --- | --- | --- | --- |
|  |  |  | **Synthetic Control** | | **Simple Average** | | **Rwanda** | | **Togo** | |
|  |  |  | **#** | **%** | **#** | **%** | **#** | **%** | **#** | **%** |
| **Sierra Leone** | 2006 | 1,173 | -95 | -8% | -98 | -8% | -15 | -1% | 11 | 1% |
|  | 2007 | 1,232 | -65 | -5% | -122 | -10% | -17 | -1% | -93 | -8% |
|  | 2008 | 1,267 | -9 | -1% | -99 | -8% | 47 | 4% | -113 | -9% |
|  | 2009 | 1,278 | 54 | 4% | -96 | -7% | 81 | 6% | -92 | -7% |
|  | 2010 | 1,315 | 141 | 11% | -92 | -7% | 105 | 8% | -91 | -7% |
|  | 2011 | 1,366 | 233 | 17% | -104 | -8% | 129 | 9% | -97 | -7% |
|  | 2012 | 1,537 | 151 | 10% | -242 | -16% | 46 | 3% | -221 | -14% |
|  | 2013 | 1,814 | 0 | 0% | -486 | -27% | -199 | -11% | -454 | -25% |
| ***Average*** |  | ***1,373*** | ***51*** | ***3%*** | ***-167*** | ***-11%*** | ***22*** | ***2%*** | ***-144*** | ***-10%*** |
| **Country** | **Year** | **Actual** | **Synthetic Control** | | **Simple Average** | | **Niger** | | **Mozambique** | |
|  |  |  | **#** | **%** | **#** | **%** | **#** | **%** | **#** | **%** |
| **Liberia** | 2006 | 501 | 58 | 12% | 575 | 115% | 269 | 54% | 267 | 53% |
|  | 2007 | 661 | -60 | -9% | 449 | 68% | 103 | 16% | 139 | 21% |
|  | 2008 | 686 | -45 | -7% | 483 | 70% | 122 | 18% | 145 | 21% |
|  | 2009 | 710 | -33 | -5% | 471 | 66% | 62 | 9% | 148 | 21% |
|  | 2010 | 736 | -11 | -2% | 487 | 66% | 70 | 9% | 154 | 21% |
|  | 2011 | 772 | 9 | 1% | 489 | 63% | 21 | 3% | 153 | 20% |
|  | 2012 | 837 | -34 | -4% | 458 | 55% | 17 | 2% | 127 | 15% |
|  | 2013 | 854 | 0 | 0% | 474 | 55% | 11 | 1% | 149 | 17% |
| ***Average*** |  | ***720*** | ***-15*** | ***-2%*** | ***486*** | ***70%*** | ***84*** | ***14%*** | ***160*** | ***24%*** |
| **Country** | **Year** | **Actual** | **Pre-outbreak Prediction Errors** | | | | | | | |
|  |  |  | **Synthetic Control** | | **Simple Average** | | **Uganda** | | **Madagascar** | |
|  |  |  | **#** | **%** | **#** | **%** | **#** | **%** | **#** | **%** |
| **Guinea** | 2006 | 1,673 | 70 | 4% | -568 | -34% | -186 | -11% | -101 | -6% |
|  | 2007 | 1,749 | -11 | -1% | -612 | -35% | -197 | -11% | -126 | -7% |
|  | 2008 | 1,781 | 11 | 1% | -591 | -33% | -126 | -7% | -91 | -5% |
|  | 2009 | 1,722 | 61 | 4% | -519 | -30% | -12 | -1% | -150 | -9% |
|  | 2010 | 1,765 | 50 | 3% | -523 | -30% | 22 | 1% | -226 | -13% |
|  | 2011 | 1,823 | 27 | 1% | -538 | -30% | 5 | 0% | -299 | -16% |
|  | 2012 | 1,889 | -2 | 0% | -581 | -31% | -65 | -3% | -362 | -19% |
|  | 2013 | 1,920 | -2 | 0% | -582 | -30% | -73 | -4% | -411 | -21% |
| ***Average*** |  | ***1,790*** | ***25*** | ***1%*** | ***-564*** | ***-32%*** | ***-79*** | ***-4%*** | ***-221*** | ***-12%*** |
| **Country** | **Year** | **Actual** | **Synthetic control** | | **Simple average** | | **Mozambique** | | **Ethiopia** | |
|  |  |  | **#** | **%** | **#** | **%** | **#** | **%** | **#** | **%** |
| **DRC** | 2003 | 545 | 4 | 1% | 443 | 81% | 106 | 19% | 71 | 13% |
|  | 2004 | 564 | -4 | -1% | 427 | 76% | 118 | 21% | 116 | 21% |
|  | 2005 | 580 | -7 | -1% | 442 | 76% | 140 | 24% | 160 | 28% |
|  | 2006 | 591 | 0 | 0% | 473 | 80% | 176 | 30% | 207 | 35% |
| ***Average*** |  | ***570*** | ***-2*** | ***0%*** | ***446*** | ***78%*** | ***135*** | ***24%*** | ***139*** | ***24%*** |
| **Country** | **Year** | **Actual** | **Synthetic Control** | | **Simple Average** | | **Burkina Faso** | | **Rwanda** | |
|  |  |  | **#** | **%** | **#** | **%** | **#** | **%** | **#** | **%** |
| **Uganda** | 1990 | 835 | -19 | -2% | -215 | -26% | -56 | -7% | 26 | 3% |
|  | 1991 | 852 | -18 | -2% | -206 | -24% | -24 | -3% | 0 | 0% |
|  | 1992 | 862 | -80 | -9% | -208 | -24% | -54 | -6% | 94 | 11% |
|  | 1993 | 893 | -83 | -9% | -239 | -27% | -80 | -9% | 36 | 4% |
|  | 1994 | 958 | -173 | -18% | -407 | -42% | -156 | -16% | -459 | -48% |
|  | 1995 | 1,015 | -222 | -22% | -370 | -36% | -190 | -19% | -340 | -34% |
|  | 1996 | 1,045 | -84 | -8% | -313 | -30% | -155 | -15% | -294 | -28% |
|  | 1997 | 1,069 | -35 | -3% | -289 | -27% | -148 | -14% | -264 | -25% |
|  | 1998 | 1,133 | -14 | -1% | -322 | -28% | -172 | -15% | -321 | -28% |
|  | 1999 | 1,171 | 0 | 0% | -322 | -28% | -178 | -15% | -366 | -31% |
| ***Average*** |  | ***983*** | ***-73*** | ***-8%*** | ***-289*** | ***-29%*** | ***-121*** | ***-12%*** | ***-189*** | ***-18%*** |

DRC, Democratic Republic of the Congo; GDP, gross domestic product.

The synthetic controls are closely matched to the actual Ebola affected countries during the pre-outbreak period, overall. Comparing each Ebola-affected country, in terms of GDP per capita, to even its two most similar countries (presented in the righthand side of the table), or to a simple unweighted average of the countries in the donor pool, reveals that the synthetic controls far more closely resemble the Ebola-affected countries. The synthetic control for Uganda is least well matched during the pre-outbreak period; however, the fact that Uganda’s actual GDP per capita exceeds the synthetic control throughout the entire pre-outbreak period suggests that any effects detected for Uganda are likely conservative lower bounds.
