# Supplementary material for: Macroeconomic impact of Ebola outbreaks in Sub-Saharan Africa and potential mitigation of GDP loss with prophylactic Ebola vaccination programs
Source: PLoS One. 2023 Apr 11;18(4):e0283721. doi: 10.1371/journal.pone.0283721 (PMC10089322; doi:10.1371/journal.pone.0283721)
Supplement: S2 Table — DRC, democratic republic of the Congo.a Includes health care practitioners. Source: (1) World Health Organization (2018); (2) Authors’ assumption; (3) World Bank (2020); (4) UNICEF (2020) and Barber and van der Weijde (2019). (DOCX) [file pone.0283721.s002.docx]

S2 Table. Incidence (per 1,000 population) by Risk Group and by Country for Year(s) of Outbreak.

| **Parameter** | **Sierra Leone** | **Liberia** | **Uganda** | **DRC** |
| --- | --- | --- | --- | --- |
| **Outbreak year(s)** | 2014-2016 | 2014-2016 | 2000 | 2007 |
| **Reported cases** |  |  |  |  |
| Overall population (1) | 14,122 | 10,675 | 425 | 264 |
| Health care workers (1)^a^ | 307 | 378 | 13 | 8 |
| Estimated cases among high-risk population^b^ | 1,707 | 2,323 | 71 | 33 |
| **Population size (% of total population)^c^** | 7,172,632 | 4,472,841 | 23,650,172 | 58,453,683 |
| Health care workers^d^ (3) | 9,730 (0.14) | 4,744 (0.11) | 38,373 (0.16) | 143,143 (0.24) |
| Frontline workers (4) | 14,345 (0.20) | 8,946 (0.20) | 47,300 (0.20) | 116,907 (0.20) |
| Armed forces (2) | 8,500 (0.12) | 2,050 (0.05) | 50,600 (0.21) | 143,000 (0.24) |
| Transportation workers (5) | 21,518 (0.30) | 13,419 (0.30) | 70,951 (0.30) | 175,361 (0.30) |
| Total high-risk population | 54,093 (0.75) | 29,158 (0.65) | 207,223 (0.88) | 578,411 (0.99) |
| **Calculated Incidence (per 1,000)** |  |  |  |  |
| High-risk population**^e^** | 31.55 | 79.69 | 0.34 | 0.06 |
| Non-high-risk population**^f^** | 1.74 | 1.88 | 0.02 | 0.004 |

DRC, Democratic Republic of the Congo.

a Due to lack of data for Uganda and DRC, the weighted average ratio of cases among health care workers to cases among the overall population in the Sierra Leone, Liberia, and Guinea outbreaks (countries for which data were available) was calculated (881 cases among health care workers / 28,606 total cases across the 3 outbreaks = 0.031) and applied to the total number of cases to estimate the number of cases among health care workers.

b Calculated by applying the incidence among health care workers to the total high-risk population.

c For countries with outbreaks spanning multiple years, the average across data years was taken.

d Includes doctors, nurses, midwives, and others employed in the hospital and community, including pharmacists, hygiene personnel, laboratory personnel, traditional medicine doctors, and community health workers.

e (number or estimated number of cases among health care workers) / (number of health care workers ÷ 1000)

f (number of cases among overall population – estimated number of cases among high-risk population) / [(population size – total high-risk population) ÷ 1000]

Sources: (1) United Nations Human Data Exchange (2019); (2) World Bank (2020); (3) World Health Organization (2018); (4) Authors’ assumption; (5) UNICEF (2020) and Barber and van der Weijde (2019).
