# Supplementary material for: Macroeconomic impact of Ebola outbreaks in Sub-Saharan Africa and potential mitigation of GDP loss with prophylactic Ebola vaccination programs
Source: PLoS One. 2023 Apr 11;18(4):e0283721. doi: 10.1371/journal.pone.0283721 (PMC10089322; doi:10.1371/journal.pone.0283721)
Supplement: S3 Table — DRC, Democratic Republic of the Congo; GDP, gross domestic product; RMSPE, root mean squared prediction error; SC, synthetic control. S3 Table reports the GDP per capita values for the Ebola-affected countries and the synthetic controls, as well as the effects in both absolute terms and percentages. Because levels of GDP per capita vary considerably across countries, the effect sizes reported as a percentage are more useful for cross-country comparisons. The placebo effect proportions associated with the effects estimated for each year and the RMSPE ratio proportions indicate that the effects are unlikely due to chance. This is particularly true for Sierra Leone and Liberia, which suffered the highest numbers of Ebola virus disease cases, and for the effects identified for the third year after each outbreak began for each country because of the apparent persistent and lagged effects. (DOCX) [file pone.0283721.s003.docx]

**S3 Table. GDP per Capita in Ebola-affected Countries and Synthetic Controls.**

| **Year** | **Sierra Leone** | | | | | **Year** | **Liberia** | | | | | **Year** | **Guinea** | | | | | | |
| --- | --- | --- | --- | --- | --- | --- | --- | --- | --- | --- | --- | --- | --- | --- | --- | --- | --- | --- | --- |
|  | **Actual** | **SC** | **Effect (US Intl. $)** | **Effect (%)** | ***Standardized Placebo Effect Proportions*** |  | **Actual** | **SC** | **Effect (US Intl. $)** | **Effect (%)** | ***Standardized Placebo Effect Proportions*** |  | **Actual** | **SC** | **Effect (US Intl. $)** | **Effect (%)** | | | ***Standardized Placebo Effect Proportions*** |
| 2014 | 1,854.23 | 1,944.24 | -90.01 | -4.6% | 0.25 | 2014 | 876.87 | 906.59 | -29.72 | -3.3% | 0.13 | 2014 | 1,945.94 | 1,957.83 | -11.90 | -0.6% | | | 0.44 |
| 2015 | 1,442.12 | 2,088.37 | -646.25 | -30.9% | 0.00 | 2015 | 935.22 | 956.48 | -21.26 | -2.2% | 0.63 | 2015 | 1,972.39 | 2,002.54 | -30.15 | -1.5% | | | 0.44 |
| 2016 | 1,500.60 | 2,186.55 | -685.95 | -31.4% | 0.00 | 2016 | 907.51 | 989.88 | -82.37 | -8.3% | 0.25 | 2016 | 2,125.08 | 2,039.45 | 85.64 | 4.2% | | | 0.33 |
| 2017 | 1,524.19 | 2,365.39 | -841.20 | -35.6% | 0.00 | 2017 | 906.71 | 1,056.58 | -149.87 | -14.2% | 0.00 | 2017 | 2,242.21 | 2,057.67 | 184.54 | 9.0% | | | 0.11 |
| *RMSPE ratio proportion* | | | | | *0.00* | *RMSPE ratio proportion* | | | | | *0.13* | *RMSPE ratio proportion* | | | | | | | *0.33* |
| **Year** | **DRC** | | | | | **Year** | **Uganda** | | | | |  |  |  |  |  |  |  |  |
|  | **Actual** | **SC** | **Effect ($)** | **Effect (%)** | ***Standardized Placebo Effect Proportions*** |  | **Actual** | **SC** | **Effect ($)** | **Effect (%)** | ***Standardized Placebo Effect Proportions*** |  |  |  |  |  |  |  |  |
| 2007 | 608.28 | 612.98 | -4.69 | -0.8% | 0.56 | 2000 | 1,186.24 | 1,160.96 | 25.28 | 2.2% | 0.00 |  |  |  |  |  |  |  |  |
| 2008 | 625.22 | 635.97 | -10.75 | -1.7% | 0.33 | 2001 | 1,247.88 | 1,264.91 | -17.03 | -1.3% | 0.25 |  |  |  |  |  |  |  |  |
| 2009 | 622.09 | 651.16 | -29.06 | -4.5% | 0.22 | 2002 | 1,291.10 | 1,337.68 | -46.58 | -3.5% | 0.25 |  |  |  |  |  |  |  |  |
| 2010 | 644.48 | 674.14 | -29.66 | -4.4% | 0.44 | 2003 | 1,323.94 | 1,381.32 | -57.38 | -4.2% | 0.25 |  |  |  |  |  |  |  |  |
| *RMSPE ratio proportion* | | | | | *0.33* | *RMSPE ratio proportion* | | | | | *0.50* |  |  |  |  |  |  |  |  |

DRC, Democratic Republic of the Congo; GDP, gross domestic product; RMSPE, root mean squared prediction error; SC, synthetic control.

S3 Table reports the GDP per capita values for the Ebola-affected countries and the synthetic controls, as well as the effects in both absolute terms and percentages. Because levels of GDP per capita vary considerably across countries, the effect sizes reported as a percentage are more useful for cross-country comparisons. The placebo effect proportions associated with the effects estimated for each year and the RMSPE ratio proportions indicate that the effects are unlikely due to chance. This is particularly true for Sierra Leone and Liberia, which suffered the highest numbers of Ebola virus disease cases, and for the effects identified for the third year after each outbreak began for each country because of the apparent persistent and lagged effects.
