# Supplementary material for: Macroeconomic impact of Ebola outbreaks in Sub-Saharan Africa and potential mitigation of GDP loss with prophylactic Ebola vaccination programs
Source: PLoS One. 2023 Apr 11;18(4):e0283721. doi: 10.1371/journal.pone.0283721 (PMC10089322; doi:10.1371/journal.pone.0283721)
Supplement: S4 Table — (DOCX) [file pone.0283721.s004.docx]

**S4 Table. Sensitivity of herd immunity factor on change in GDP per Capita effect estimates.**

| **Country** | **Outbreak Year** | **Actual** | **Averted Cases** | | | | **Share of Actual Impact Mitigated by Vaccine (%)** | | | |
| --- | --- | --- | --- | --- | --- | --- | --- | --- | --- | --- |
|  |  | **Total Reported Ebola Cases** | **Low** ^a^  **(-10%)** | **Low**  **(+10%)** | **High** ^b^  **(-10%)** | **High**  **(+10%)** | **Low**  **(-10%)** | **Low**  **(+10%)** | **High**  **(-10%)** | **High**  **(+10%)** |
| Sierra Leone | 2014 | 14,122 | 7,880 | 9,631 | 11,566 | 14,122 | 78.05 | 82.63 | 85.85 | 100.00 |
| Liberia | 2014 | 10,675 | 5,957 | 7,280 | 8,743 | 10,675 | 75.21 | 81.48 | 86.40 | 100.00 |
| Uganda | 2000 | 425 | 237 | 290 | 348 | 425 | 1.52 | 1.84 | 2.20 | 100.00 |
| DRC | 2007 | 264 | 147 | 180 | 216 | 264 | 0.86 | 1.05 | 1.26 | 100.00 |

DRC, Democratic Republic of the Congo; GDP, gross domestic product; RMSPE, root mean squared prediction error; SC, synthetic control.

^a^ Low coverage/efficacy: 60% efficacy, 30% coverage of high-risk population plus 5% coverage of non-high-risk population. The estimated impact of this vaccination scenario on the cumulative number of Ebola cases was assumed to be 62%, based on the estimated percentage of cumulative cases averted versus no vaccination as reported in Table 14 for vaccinating 30% of the high-risk population and 5% of the general population in the 2018 outbreak in the North-Kivu province of the DRC [SmartAnalyst Model, Impact of prophylactic vaccination strategies on Ebola virus transmission: Model enhancement and evaluation of vaccination strategies. Ed. 0.2 2020. 15 July 2020.] Those values were for the baseline scenario and assumed no reduced infectiousness from vaccination.

^b^ High coverage/efficacy: 90% efficacy, 60% coverage of high-risk population plus 10% coverage of non-high-risk population. The estimated impact of this vaccination scenario on the cumulative number of Ebola cases was assumed to be 91%, based on the estimated percentage of cumulative cases averted versus no vaccination as reported in Table 24 for vaccinating 60% of the high-risk population and 10% of the general population in the 2018 outbreak in the North-Kivu province of the DRC [SmartAnalyst Model, Impact of prophylactic vaccination strategies on Ebola virus transmission: Model enhancement and evaluation of vaccination strategies. Ed. 0.2 2020. 15 July 2020.] Those values were for the upside scenario and assumed no reduced infectiousness from vaccination.
