# Supplementary figures and images for: Macroeconomic impact of Ebola outbreaks in Sub-Saharan Africa and potential mitigation of GDP loss with prophylactic Ebola vaccination programs
Source: PLoS One. 2023 Apr 11;18(4):e0283721. doi: 10.1371/journal.pone.0283721 (PMC10089322; doi:10.1371/journal.pone.0283721)

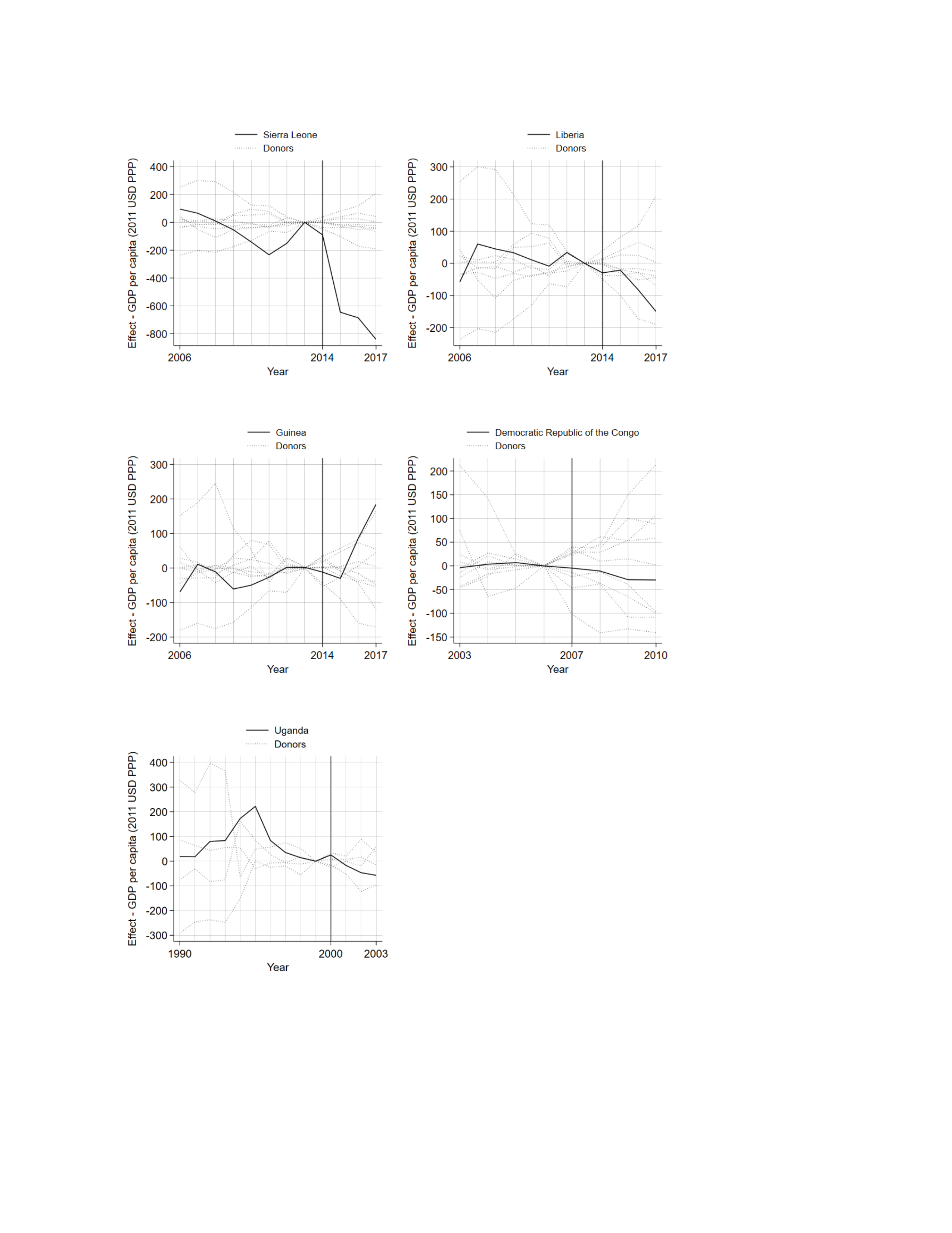

Supplement: S1 Fig — plots the prediction errors in the pre-outbreak period and effects in the post-outbreak period for the Ebola-affected country. The prediction errors and the placebo effects are plotted for the comparison countries. The outbreak is represented by the vertical line. The ratio of the effect sizes in the post-outbreak period to the size of the prediction errors in the pre-outbreak period were generally much greater for the Ebola-affected countries than for the donor countries that received the placebo treatment. The effect sizes are generally larger than the placebo effects as well. (TIFF) [file pone.0283721.s005.tiff]

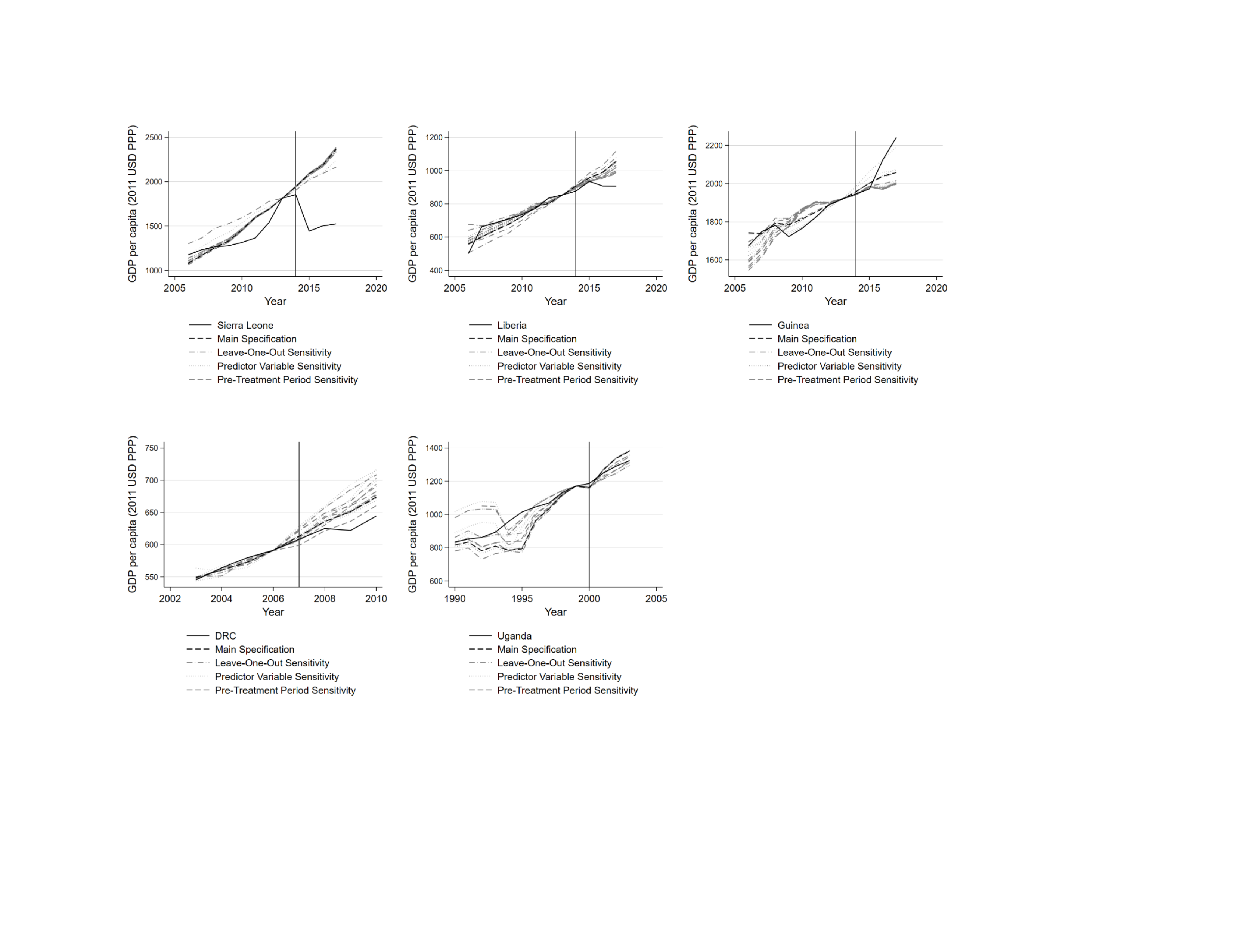

Supplement: S2 Fig — graphs the actual GDP per capita of the Ebola-affected countries against the full time series of the alternative specifications alongside the main synthetic control specification. Alternative predictor variables that were added or substituted include capital stock, population density, inflation, electrification, and government expenditure on health care. (TIFF) [file pone.0283721.s006.tiff]
