# Supplementary material for: Macroeconomic impact of Ebola outbreaks in Sub-Saharan Africa and potential mitigation of GDP loss with prophylactic Ebola vaccination programs
Source: PLoS One. 2023 Apr 11;18(4):e0283721. doi: 10.1371/journal.pone.0283721 (PMC10089322; doi:10.1371/journal.pone.0283721)
Supplement: S1 File — (DOCX) [file pone.0283721.s007.docx]

**S1 Equation. The RMSPE ratio.**

The root mean square prediction error (RMSPE) for the unit in the pre-event period is defined as

$$Pre-RMSPE= \left( \frac{1}{T_{0}}\sum_{t=1}^{T_{0}} \left( Y_{1t}-\sum_{j=2}^{J+1} w^{*}Y_{jt} \right)^{2} \right)^{\frac{1}{2}}.$$

The RMSPE for the unit in the postintervention period is defined as

$$Post-RMSPE= \left( \frac{1}{T}\sum_{t=T_{0}}^{T} \left( Y_{1t}-\sum_{j=2}^{J+1} w^{*}Y_{jt} \right)^{2} \right)^{\frac{1}{2}}.$$

The RMSPE ratio for the unit is

$$RMSPE Ratio=\frac{Post-RMSPE}{Pre-RMSPE}.$$

The proportion of RMSPE ratios generated from the placebo tests that were at least as large as the RMSPE ratio of the Ebola-affected country are presented in S3 Table as RMSPE ratio proportions.
